# ST6GALNAC4 promotes hepatocellular carcinogenesis by inducing abnormal glycosylation

**DOI:** 10.1186/s12967-023-04191-7

**Published:** 2023-06-29

**Authors:** Da Man, Yifan Jiang, Deguo Zhang, Jingjing Wu, Bo Ding, Hanqing Liu, Guangming Xu, Jiahua Lu, Junnan Ru, Rongliang Tong, Shusheng Zheng, Diyu Chen, Jian Wu

**Affiliations:** 1 Division of Hepatobiliary and Pancreatic Surgery, Department of Surgery, The First Affiliated Hospital, Zhejiang University School of Medicine, Hangzhou, 310003 Zhejiang China; 2 NHC Key Laboratory of Combined Multi-organ Transplantation, Hangzhou, 310003 Zhejiang China; 3 Key Laboratory of the diagnosis and treatment of organ Transplantation, Research Unit of Collaborative Diagnosis and Treatment for Hepatobiliary and Pancreatic Cancer, Chinese Academy of Medical Sciences (2019RU019), Hangzhou, 310003 Zhejiang China; 4 Key Laboratory of Organ Transplantation, Research Center for Diagnosis and Treatment of Hepatobiliary Diseases, Hangzhou, 310003 Zhejiang China; 5 State Key Laboratory for Diagnosis and Treatment of Infectious Diseases, National Clinical Research Center for Infectious Diseases, National Medical Center for Infectious Diseases, Collaborative Innovation Center for Diagnosis and Treatment of Infectious Diseases, The First Affiliated Hospital, Zhejiang University School of Medicine, Hangzhou, China

**Keywords:** Hepatocellular carcinoma, ST6GALNAC4, Immunosuppressive, Galectin3, Glycosylation

## Abstract

Hepatocellular carcinoma (HCC) is one of the most lethal tumor types worldwide. Glycosylation has shown promise in the study of tumor mechanisms and treatment. The glycosylation status of HCC and the underlying molecular mechanisms are still not fully elucidated. Using bioinformatic analysis we obtained a more comprehensive characterization of glycosylation of HCC. Our analysis presented that high glycosylation levels might correlate with tumor progression and poor prognosis. Subsequent Experiments identified key molecular mechanisms for ST6GALNAC4 promoting malignant progression by inducing abnormal glycosylation. We confirmed the contribution of ST6GALNAC4 to proliferation, migration, and invasion in vitro and in vivo. Mechanistic studies revealed that ST6GALNAC4 may be induced abnormal TGFBR2 glycosylation, resulting in the higher protein levels of TGFBR2 and TGF$\beta$ pathway increased activation. Our study also provided a further understand of immunosuppressive function of ST6GALNAC4 through T antigen-galectin3+ TAMs axis. This study has provided one such possibility that galectin3 inhibitors might be an acceptable treatment choice for HCC patients with high T antigen expression.

**Supplementary Information:**

The online version contains supplementary material available at 10.1186/s12967-023-04191-7.

## Introduction

liver cancer caused 830,180 deaths in 2020, and is now the 3rd leading cause of cancer-related death worldwide. Hepatocellular carcinoma (HCC) is the leading type of liver cancer, accounting for about 80% of all primary liver cancers [1, 2]. Although early detection and newer therapies has been associated with improved overall survival, disparities in outcomes of care for HCC persist [3]. Tumor heterogeneity has been documented in multiple tumors including HCC [4]. To explain the disparity, the difference among tumor genotypes are likely play a role [5]. Therefore, to developing targeted therapy and precision medicine, accurate molecular subtyping of HCC is crucial.

Glycosylation is the most common post-translational modification medicated by specific enzymes [6]. Glycoproteins are initiated with the linkage of glycans covalently to polypeptide backbone via nitrogen(N-linked) or oxygen(O-linked) [7, 8].

Glycosylation is an enzymatic process involving several specific enzymes, called glycosyltransferases. Abnormal glycosylation is usually associated with aberrant glycosyltransferases expression in cancer. Previous studies have noted aberrant glycosyltransferases expression in HCC, which is associated with poor prognosis. Given the diversity and heterogeneity of glycosyltransferases in HCC, better understanding of glycosyltransferases, particularly glycosyltransferases related to O-glycosylation, may lead to an entirely new angle with cancer therapy.

ST6GALNAC4 is a member of the sialyltransferases, which catalyzes the transfer of sialic acid from cytidine monosphosphate (CMP)-sialic acid to galactose-containing substrates [9]. However, Elevated levels of T-Antigen results from capping of the motif by ST6GALNAC4 had been observed across tumors [10]. ST6GALNAC4 was reported to influence patient prognosis though subverting immunosurveillance in Chronic lymphocytic leukemia [11]. The relationship with poor prognosis was also found in Uterine corpus endometrial carcinoma [12] and thyroid carcinoma [13]. However, detailed roles of ST6GALNAC4 in HCC remain obscure.

In this study, we found that activation of ST6GALNAC4 in HCC may have a significant role in malignancies among numerous glycosyltransferases. Increased ST6GALNAC4 could stimulate tumor cell proliferation, migration, and immunosuppression. Mechanistically, High expression of ST6GALNAC4 may drive high expression levels of TGFBR2 to promote proliferation, and invasion. Concurrently, High expression of ST6GALNAC4 may be associated with recruiting galectin3+ TAMs through T antigen, in turn aiding with tumor immunosuppression.

## Method

### Patients and tissue samples

Tumor and adjacent non-tumor tissues were collected from patients undergoing surgery for HCC at the First Affiliated Hospital, Zhejiang University School of Medicine, Zhejiang, China. 90 HCC tumor samples and the adjacent non-tumor tissues with clinicopathological and follow-up data were used for tissue microarray. This research was approved by the Ethical Review Committee of this hospital. Written informed consent was received according to the guidelines of the Declaration of Helsinki. The demographic and clinical characteristics of included patients is in the additional file 1: Table S1).

### Bioinformatics analysis

The Cancer Genome Atlas (TCGA) dataset was selected, which contained transcript expression data and corresponding clinical information from TCGA (www.tcga-data.nci.nih.gov/tcga). Counts were converted to units of TPM, followed by transforming to log2(TPM + 1). The least absolute shrinkage and selection operator (LASSO) regression [14] algorithm was used for feature selection. The R package glmnet was used for the analysis. For Kaplan–Meier survival analysis [15] of the risk model, Log-rank tests were used to compare between-group differences in survival curves. Using The timeROC(v 0.4) analysis, we compared the predictive accuracy of genes and risk score. Plots were generated with the ggplot2 package. The scRNA-seq data was obtained from GSE149614 in GEO database (https://www.ncbi.nlm.nih.gov/geo/query/acc.cgi?acc=GSE149614) submitted by Lu et al. CellRanger (v6.0.2) was used to read mapping and gene expression quantification. Cells with less than 1000 UMIs or $>15\%$ mitochondria genes were excluded. Doublets were assessed using the DoubletFinder (v2.0.3) algorithm each sample. We used Harmony to remove batch effect described by Korsunsky et al. [16]. Visualizations were generated using Uniform Manifold Approximation and Projection (UMAP). Then we performed louvain clustering by setting the parameter resolution = 2 to achieve 13 clusters. First each cluster was annotated based on the classical marker genes. We also referred cluster specific DEGs identified by “FindAllMarkers” function using Wilcoxon Rank Sum test in Seurat R package.We provide the code for the bioinformatics analysis in the Additional file 2: Code.

### Statistical analysis

Experiments were repeated at least three times, mean values ± SD are shown. Statistical analyses were performed using GraphPad Prism 8.0 (GraphPad Software). Two-tailed unpaired or paired Student’s t test was applied for comparison. Kaplan–Meier method and log-rank test were used for survival analysis. Linear regression analyses were performed to determine correlation between two variables. P < 0.05 was regarded as statistically significant (*P < 0.05; **P < 0.01; ***P < 0.001; ****P < 0.0001. ns, not significant). Further details of materials and methods are provided in the Additional file 3: Supplementary methods.

## Results

### Construction and validation of prognostic signatures by O-glycosylation

**Fig. 1:**
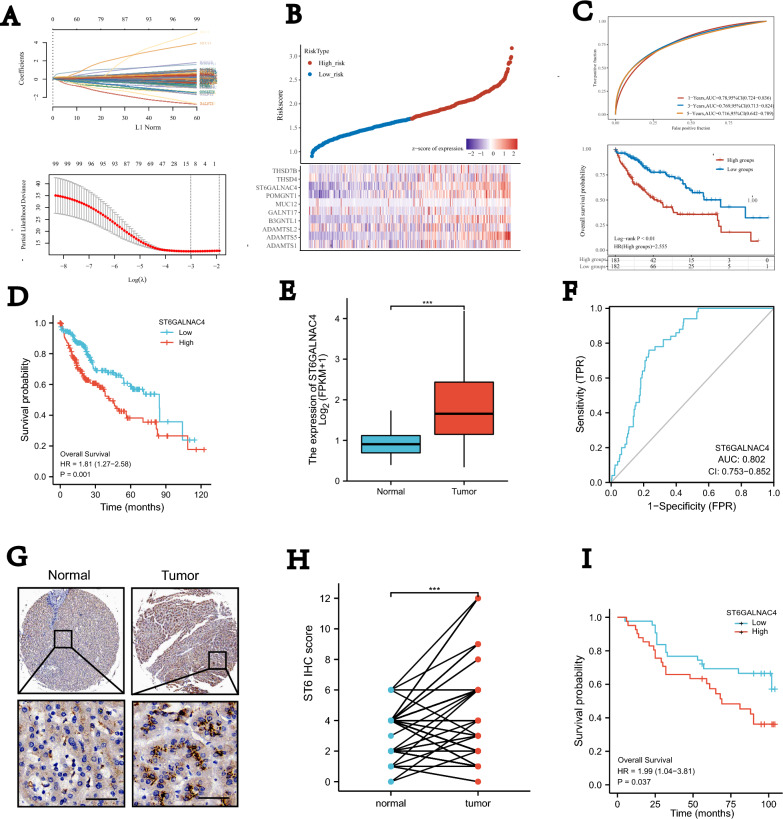
Identification of key glycosyltransferase related to O-glycosylation indicating poor prognosis in HCC. **A** Partial likelihood deviance of different combinations of variables calculated via the LASSO Cox regression model and LASSO coefficient profiles of candidate genes. **B** Heatmap of the expression levels of candidate genes. **C** ROC AUC of the regression model and KM curves of different groups. **D** KM curves of ST6GALNAC4 in TCGA data sets. **E** Expression of ST6GALNAC4 in TCGA data sets. **F** ROC curve analysis of ST6GALNAC4 diagnosis. **G** IHC staining of ST6GALNAC4 in HCC and adjacent liver tissue (scale bar, 50 $\mu$m; magnification, ×400). **H** IHC score of ST6GALNAC4 in 90 pairs of our own HCC tissue array. **I** KM curves of ST6GALNAC4 in our own HCC tissue array. *P < 0.05; **P < 0.01; ***P < 0.001; ****P < 0.0001. ns, not significant

In order to screen out a key glycosyltransferase related to O-glycosylation indicating poor prognosis in HCC, 111 glycosyltransferases in the gene set named “REACTOME_O_LINKED_GLYCOSYLATION” were retrieved from the Molecular Signatures Database (MSigDB, http://www.gsea-msigdb.org/gsea/msigdb/). These candidate genes were used in LASSO regression analysis (Fig. 1A). Finally, 13 genes were used to construct a prognostic model. The following formula was utilized: O-glycosylation score=($-$0.072* expression level of ADAMTS1)+($-$0.0081* expression level of ADAMTS10)+(0.3984* expression level of ADAMTS5)+($-$0.0152* expression level of ADAMTSL2)+(0.091* expression level of B3GNTL1)+($-$0.0293* expression level of GALNT16)+($-$0.0775* expression level of GALNT17)+($-$0.0651* expression level of GALNT8)+($-$0.0205* expression level of MUC12)+(0.1897* expression level of POMGNT1)+(0.1264* expression level of ST6GALNAC4)+($-$0.0557* expression level of THSD4)+($-$0.0241* expression level of THSD7B). Then we assessed the formula. The formula was used to produce a O-glycosylation score for each sample. Samples were ranked by the O-glycosylation score and separated into two groups with the median cutoff score (Fig. 1B). The ROC curves to predict risk of death at years 1, 3, and 5 were plotted in Fig. 1C. Kaplan–Meier survival curves showed a significant trend in overall survival probability between two group (Fig. 1C).

### The identification of key glycosyltransferase related to O-glycosylation

Combination with the prognostic information in TCGA (Additional file 4: Fig. S1),we narrowed our focus to the ST6GALNAC4 as a candidate to mediate the glycosylation in HCC. In order to clarify the role of ST6GALNAC4 in HCC, TCGA LIHC transcriptomic data sets were used to compare levels of expression of HCC and normal tissues. These results indicate that ST6GALNAC4 expression is highly enriched in HCC compared with that in the normal tissues (Fig. 1E). Immunohistochemical (IHC) staining (Additional file 5 Fig. S2) in 90 pairs of our own HCC specimens also demonstrated that ST6GALNAC4 highly upregulated in HCC (Fig. 1G, H). Cox regression model with ROC curve analysis was performed to confirm that high expression of ST6GALNAC4 was predictors of tumor (AUC = 0.802; Fig. 1F). The two independent datasets, TCGA and our own specimens, demonstrated ST6GALNAC4 predicted a significantly worse overall survival (Fig. 1D, I).

### ST6GALNAC4 promotes HCC cell proliferation, migration, and invasion in vitro

**Fig. 2:**
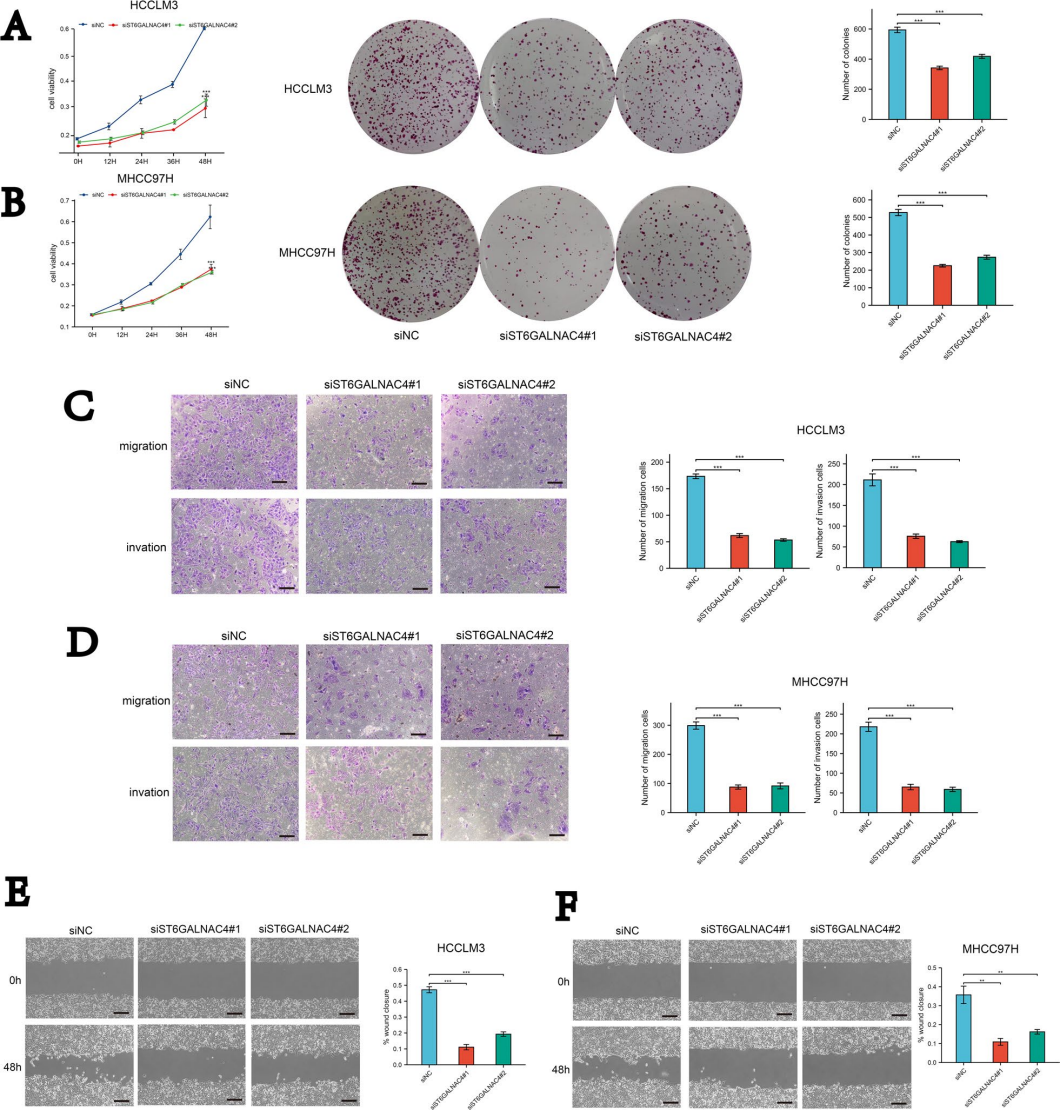
Knockdown of ST6GALNAC4 attenuated HCC cell proliferation, migration, and invasion in vitro. We used small interfering RNAs (siRNAs) to knock down ST6GALNAC4 expression in HCC cells. ST6GALNAC4 knockdown resulted in the reduced proliferative capacity of HCCLM3 (**A**) and MHCC97H (**B**) detected by CCK-8 and colony formation assay. ST6GALNAC4 knockdown resulted in impaired migratory and invasive capabilities of HCCLM3 (**C**, **E**) and MHCC97H (**D**, **F**). scale bar, 200 $\mu$m; magnification, ×100. *P < 0.05; **P < 0.01; ***P < 0.001; ****P < 0.0001. ns, not significant

We next investigated the effect of ST6GALNAC4 on proliferation, migration, and invasion in HCCLM3 and MHCC97H cells lines. Cell proliferation of HCC cell lines was detected using the CCK8 (Fig. 2A, B). We found that knockdown of ST6GALNAC4 (using siRNA) reduced cell proliferation. Knockdown of ST6GALNAC4 also significantly reduced colony formation. The transwell assays (Fig. 2C, D) and wound-healing (Fig. 2E, F) assays revealed that knockdown of ST6GALNAC4 exhibited a markedly weakened migration and invasion abilities. As observed for ST6GALNAC4 stable overexpression cell lines, it was the opposite. high expression of ST6GALNAC4 enhanced the cell proliferation, migration, and invasion abilities in vitro (Additional file 6: Fig. S3).

### ST6GALNAC4 promotes HCC cell proliferation and in vivo

**Fig. 3:**
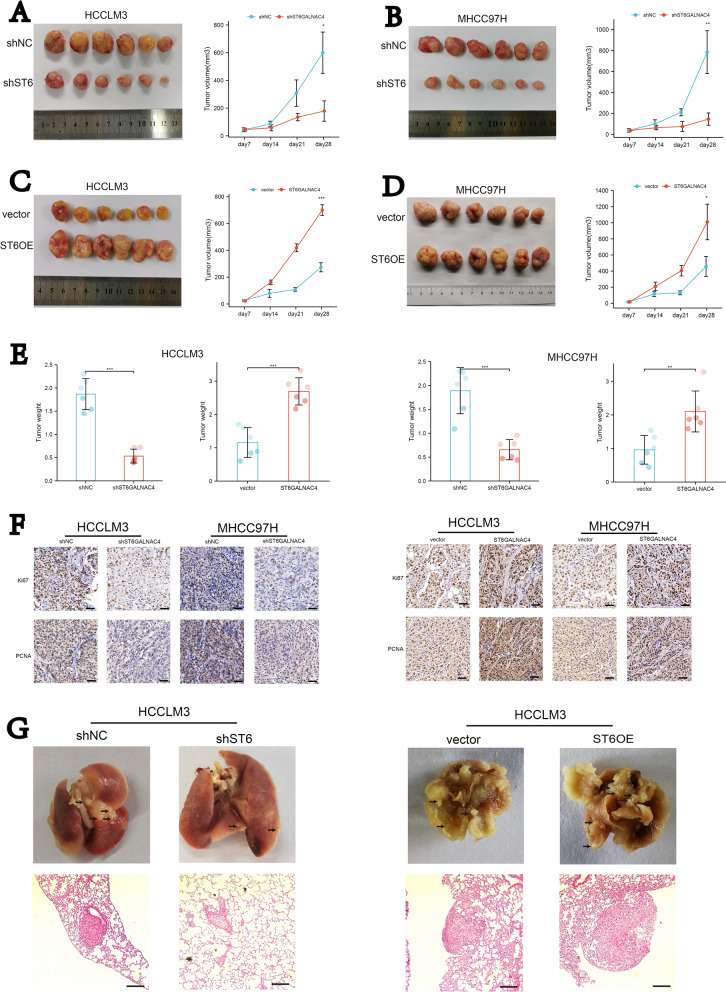
ST6GALNAC4 enhanced HCC cells proliferation, migration, and invasion in vivo. Tumor growth curve of stable ST6GALNAC4 knockdown HCCLM3 (**A**) and MHCC97H (**B**) in subcutaneous xenografted nude mice models compared with control. Tumor growth curve of stable ST6GALNAC4 overexpressing HCCLM3 (**C**) and MHCC97H (**D**) in subcutaneous xenografted nude mice models compared with control. **E** Tumor weights in subcutaneous xenografted nude mice models were recorded. **F** Tumors in different group were staining with Ki67 and PCNA. scale bar, 50 $\mu$m; magnification, ×200. **G** Pulmonary metastasis models were constructed with stable ST6GALNAC4 knockdown or overexpressing HCCLM3 cell compared with control. Staining with hematoxylin and eosin revealed tumors. scale bar, 50 $\mu$m; magnification, ×200. *P < 0.05; **P < 0.01; ***P < 0.001; ****P < 0.0001. ns, not significant

Next, we used established stable ST6GALNAC4 knockdown cell lines (using lentiviral shRNAs) and stable ST6GALNAC4 overexpression cell lines to investigated the contributions of ST6GALNAC4 to tumor proliferation and invasiveness in vivo. Subcutaneous xenografted in nude mice models of above cell lines were established. Tumors in shST6GALNAC4 group were visible decreased compared to the control group (Fig. 3A, B). While overexpression of ST6GALNAC4 reversed this trend (Fig. 3C, D). Significant differences in tumor weight between groups are presented in Fig. 3E. These tumors were also analyzed for Ki67 and PCNA expression using IHC (Fig. 3F). We then established the lung metastasis model intravenously injecting the control and ST6GALNAC4 knockdown or overexpression HCCLM3 cells via the tail vein. However, the number of lung metastases was significantly reduced in shST6GALNAC4 group while ST6GALNAC4 overexpression group shown the opposite trend (Fig. 3G). All these vivo results also supports the conclusion that ST6GALNAC4 could be identified with pro-invasive and pro-tumorigenic functions.

### ST6GALNAC4 increases protein levels of TGFBR2 at the posttranslational levels by glycosylation

**Fig. 4:**
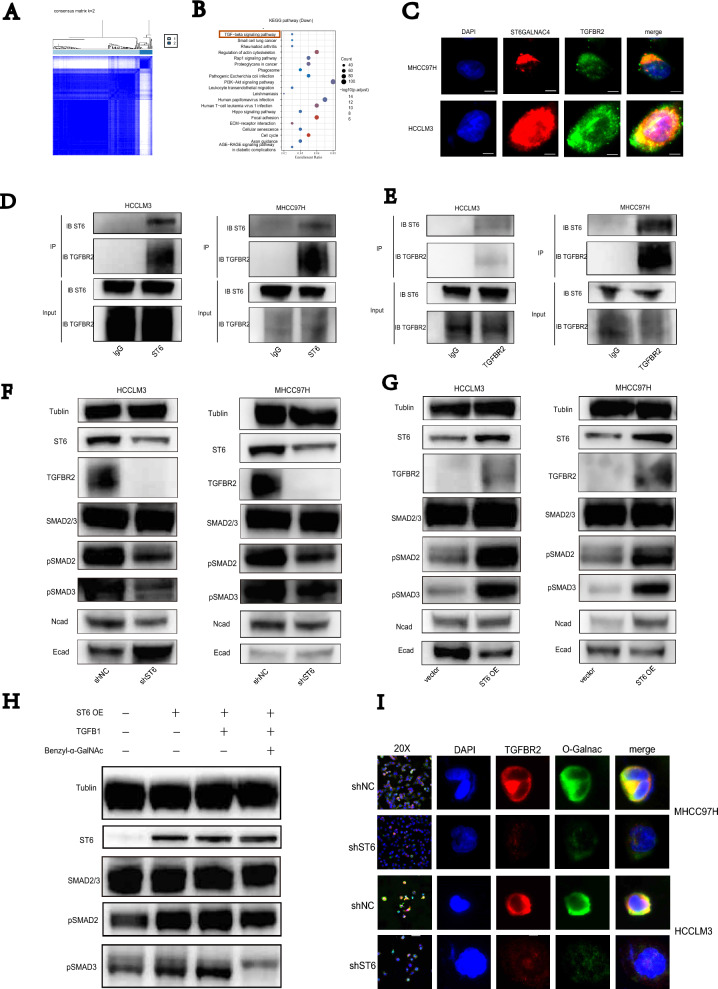
ST6GALNAC4 might regulate TGFBR2 expression by elevated glycosylation level in HCC. **A** heatmap of hierarchical clustering by O-glycosylation state samples. **B** Enrichment analysis using the KEGG database. **C** Co-localization of ST6GALNAC4 and TGFBR2 in infected HCCLM3 and MHCC97H cells by immunofluorescence. scale bar, 5 $\mu$m; magnification, ×2000. **D** ST6GALNAC4 antibodies pulled down TGFBR2 protein in coIP experiments. **E** TGFBR2 antibodies pulled down ST6GALNAC4 protein in coIP experiments. **F** Effect of ST6GALNAC4 knockdown on TGFBR2,SMAD2/3, pSMAD2, pSMAD3, N-cadherin, E-cadherin, by western blotting, $\beta$-tubulin were used as internal standards. **G** Effect of ST6GALNAC4 overexpression on TGFBR2, SMAD2/3, pSMAD2, pSMAD3, N-cadherin, E-cadherin by western blotting, $\beta$-tubulin were used as internal standards. **H** Effect of TGFB1 Protein and Benzyl-$\alpha$-GalNAc in ST6GALNAC4 overexpression cells on SMAD2/3, pSMAD2, pSMAD3 by western blotting, $\beta$-tubulin were used as internal standards. **I** Effect of ST6GALNAC4 knockdown on TGFBR2 and O-GalNAcylation by immunofluorescence. scale bar, 5 $\mu$m; magnification, ×2000

We further interrogated the mechanism. We used TCGA HCC samples and grouped two distinct O-glycosylation state samples using the ConsensusClusterPlus package [17]. Previously mentioned 111 glycosyltransferases related to O-glycosylation were used as the definition of gene expression features (Fig. 4A). We then performed GO and KEGG enrichment analysis for differential gene expression ($|LogFC| > 1.5$). Transforming growth factor $\beta$,(TGF$\beta$)were enriched in the top position in KEGG enrichment analysis (Fig. 4B). Varying components in TGF$\beta$ signaling pathway, especially TGF$\beta$ receptor type-1 and 2, can be glycosylated that resulted in functional changes [18]. To investigate this possibility, we sought to validate the ST6GALNAC4 interaction with TGFBR1 and 2 using coIP. As expected, ST6GALNAC4 antibody could pull down TGFBR2 protein while TGFBR2 antibody could also pull down ST6GALNAC4 (Fig. 4D, E). Immunofluorescence staining further indicated co-localization of ST6GALNAC4 with TGFBR2 (Fig. 4C). We further validated knock-down ST6GALNAC4 decreased TGFBR2 protein levels. Moreover, ST6GALNAC4 depletion decreased pSMAD2/3 levels. As the classic TGF$\beta$ downstream epithelial-mesenchymal transitions (EMT) genes, N-cadherin decreased while E-cadherin elevated while ST6GALNAC4 were knock-down (Fig. 4F). The opposite trend was observed while we overexpressed ST6GALNAC4 (Fig. 4G). We also identified positive interrelationship between O-GalNAcylation levels and ST6GALNAC4. The degree of co-localization between TGFBR2 and O-GalNAc was significantly lower in ST6GALNAC4 knockdown cells (Fig. 4I). TGFB1 Protein and Benzyl-$\alpha$-GalNAc(O-galnac inhibitor) were then used to examine the impact of O-glycosylation on the TGFβ pathway in HCC. In Fig. 4H, TGFβ pathways upregulation by TGFB1 Protein can be reversed with Benzyl-$\alpha$-GalNAc (Fig. 4H). Combined the above results suggested that ST6GALNAC4 might regulate TGFBR2 protein levels at the posttranslational level by glycosylation.

### ST6GALNAC4 leads to the depletion of CD8+ cells in HCC

**Fig. 5:**
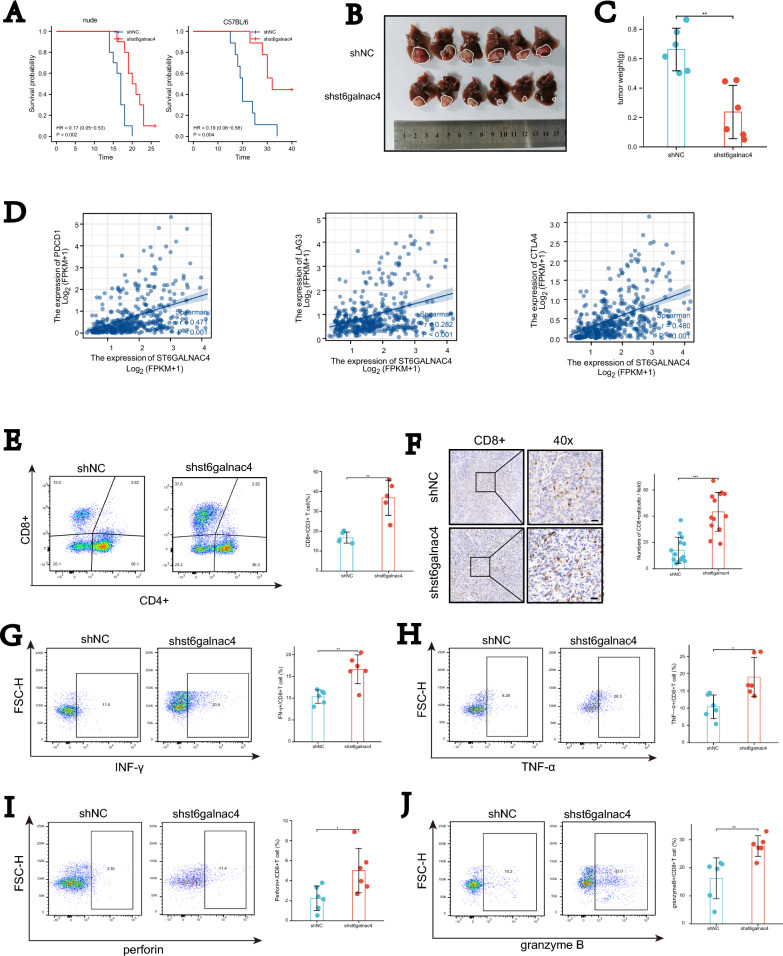
ST6GALNAC4 exerted an immunosuppressive effect by inducing CD8+ T cell depletion in HCC. **A** KM curve in orthotopic liver model in both nude mice and C57BL/6 mice with shst6galnac4 and shNC hep1-6 cells. **B** Tumors in orthotopic liver model in C57BL/6 mice. **C** Tumor weights in orthotopic liver model in C57BL/6 mice were recorded. **D** Correlation between the expression of ST6GALNAC4 and exhaustion markers of T cells in TCGA data set. **E** Proportions of CD8+ and CD4+ T cell populations between shst6galnac4 group and control group on flow cytometry. **F** CD8 immunohistochemistry in tumors between shst6galnac4 group and control group on flow cytometry. Proportions of INF-$\gamma$ (**G**), TGF-$\alpha$ (**H**), perforin (**I**), and Granzyme B (**J**)+ T cell populations between shst6galnac4 group and control group on flow cytometry. *P < 0.05; **P < 0.01; ***P < 0.001; ****P < 0.0001. ns, not significant

As glycosylase-glycosylated antigens-lectin receptors axis plays unique immunoregulatory roles on the tumor microenvironment [10, 19, 20], we proposed a hypothesis that ST6GALNAC4 might exert immunosuppressive roles in HCC. We next evaluated the immune function of ST6GALNAC4 using an orthotopic tumor mouse model. We knocked down st6galnac4 using shRNA in mouse hepatoma cell line (Hepa1-6). We then implanted Hepa1-6 to generate orthotopic liver model in both nude mice and C57BL/6 mice. We found that shst6galnac4 group had a better prognosis in nude mice, consistently with our previous experiment. It is noteworthy that the difference in the survival was much more pronounced between the two samples in C57BL/6 mice, suggesting a possible immunosuppressive role for HCC (Fig. 5A–C). Since the correlations between the ST6GALNAC4 expression and exhaustion markers of T cells in TCGA (Fig. 5D), we explored the causal relationship between the two. Using the orthotopic model mentioned earlier, we observed increased proportion of CD8+ T cells in shst6galnac4 group (Fig. 5E). The CD8 immunohistochemistry also confirmed the same (Fig. 5F). Granzyme B (GZMB), perforin (PRF), INF-$\gamma$, and TNF-$\alpha$ expressing cells were also significantly higher in CD8+ T cells in shst6galnac4 group (Fig. 5G–J).

### ST6GALNAC4 is associated with higher T antigen expression in HCC

We further interrogated the mechanism that ST6GALNAC4 leads to immunosuppression. As one of the most attractive O-glycans targets for anticancer therapy, T antigen is associated with immunosuppression in multiple malignancies [21]. The level of T antigen at the tumor is has been largely unstudied in HCC. First, we verified highly upregulated T antigen in HCC by immunohistochemistry with peanut agglutinin (PNA), which was a Gal-GalNAc specific lectin (Additional file 7: Fig. S4A). The same conclusion was supported in immunofluorescence using fluorescein isothiocyanate (FITC)-labeled PNA and Western blots (Additional file 7: Fig. S4B, C).Then the result of Western blot validated the contribution of ST6GALNAC4 in promoting the high levels of T antigen in HCC (Additional file 7: Fig. S4D).

### Galectin3 in TAMs acts as a primary lectin receptor responding to elevated T antigen in HCC

**Fig. 6:**
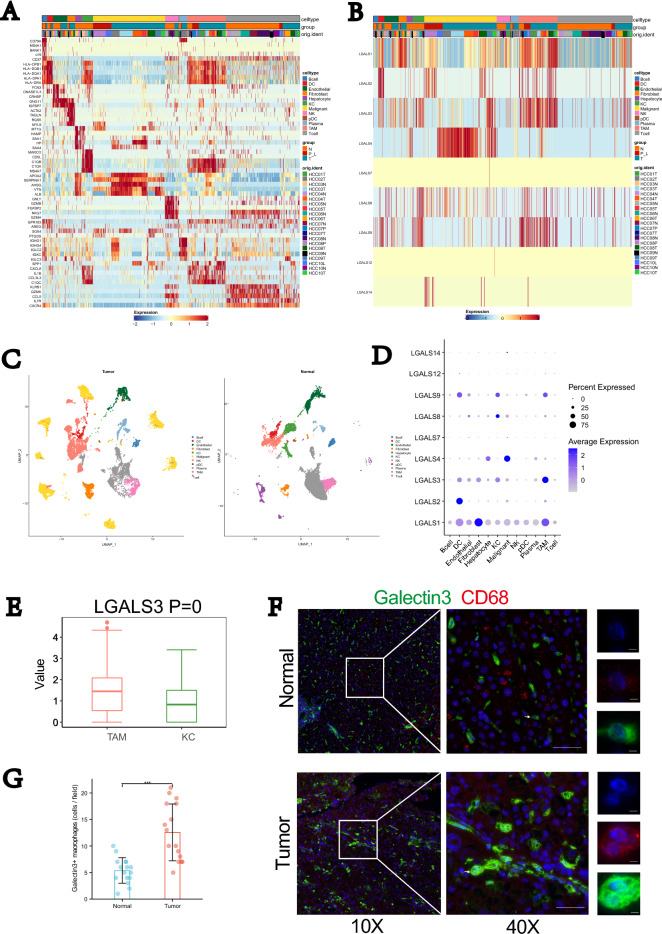
Identification of possible galectins response to T antigen in HCC through single-cell atlas. **A** Heatmap of canonical cell-type markers of 12 major cell types. **B** Heatmap of expression of members of Lgals (galectin3) family. **C** UMAP visualization of cancer cell clusters. **D** Dot Plot of expression of Lgals (galectins) in different cell types. **E** Expression of LGALS3(galectin3) between TAMs in tumors tissue and Kuppfer cells in normal tissue. **F** Co-localization of galectin3 and CD68 in tumors tissue and in normal tissue by immunofluorescence. Scale bar, 25 $\mu$m; magnification, ×400. **G** Numbers of galectin3+ macrophages in tumors tissue and in normal tissue. *P < 0.05; **P < 0.01; ***P < 0.001; ****P < 0.0001. ns, not significant

The possible mechanisms were further explored. Some lectin receptors, like sialic acid-binding immunoglobulin-like lectins (siglecs) and galactose-specific lectin(galectins), are expressed at immune cell types. These receptor are able to respond to tumor glyco-code [22]. Current studies had showed galectins play a prominent role in a wide range of diseases, including tumors [23]. We assumed that ST6GALNAC4 might recruit immunosuppressive cells through glycosylated antigen and specific galectin. To this end, we used a publicly available single-cell data set (GSE149614) to provide specific receptors information at immune cells. Following gene filtering and normalization, we performed uniform manifold approximation and projection (UMAP), which identified 12 clusters (Fig. 6A). Next, we analyzed the expression levels of galectins (alias Lgals) across different cell types (Fig. 6B). We found a certain level of galectin1,2,3,4,8,9,14 in different cell types. It is noteworthy that, Compared with certain degree of galectin1 in almost all cells, galectin3 expression is significantly elevated in TAMs (Fig. 6D, E). We therefore focused our analyses in galectin3+TAMs. Immunofluorescence analyses in HCC tumor tissues and respective normal tissues supported above results (Fig. 6F, G).

### Galectin3+TAMs promotes tumor growth by inhibiting CD8+T cell infiltration

**Fig. 7:**
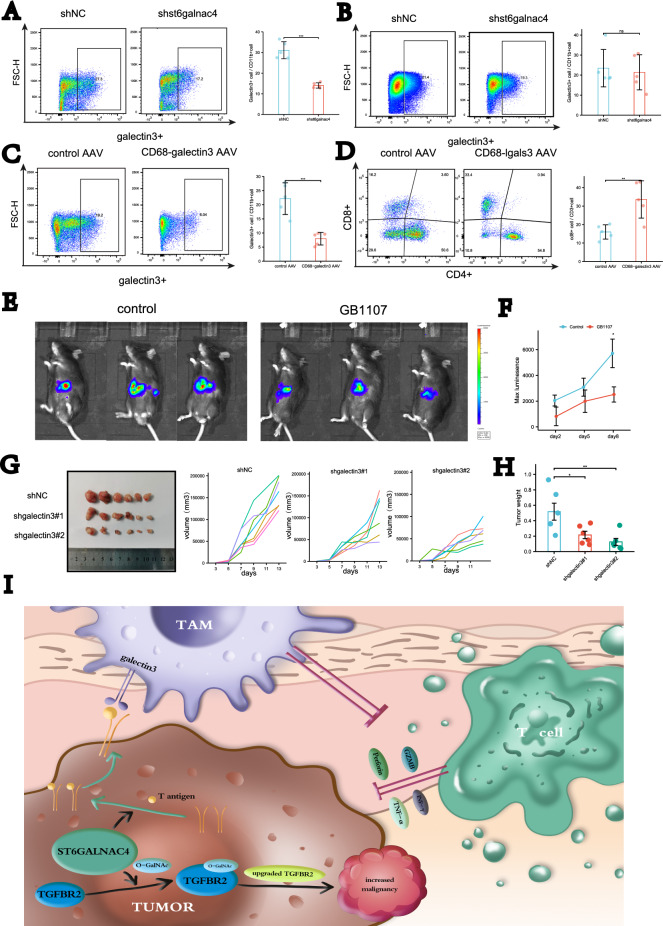
ST6GALNAC4 exerted immunosuppressive effects through the recruitment of Galectin3+TAMs. Proportions of Galectin3+ macrophages in tumor (**A**) and spleen (**B**) between shst6galnac4 group and control group on flow cytometry. **C** Proportions of Galectin3+ macrophages in spleen between shLgals3 AAV infection group and control group on flow cytometry. **D** Proportions of CD8+ and CD4+ T cell populations between shLgals3 AAV infection group and control group on flow cytometry. **E** Tumors in orthotopic liver model in C57BL/6 mice between GB1107 treatment and control group in vivo imaging system (IVIS). **F** Maximum fluorescence curve between GB1107 treatment and control group in vivo imaging system (IVIS). **G** Tumors volume curve in subcutaneous xenografted model in C57BL/6 mice between shLgals3 group and control group, each line indicates a single mouse. **H** Tumors weight in cell mixing subcutaneous xenografted model in C57BL/6 mice between shLgals3 group and control group. **I** Schematic representation of the mechanism for ST6GALNAC4 in HCC

As previously, galectin3(Lgals3) had proved to increase transcription in TAMs. To determine whether galectin3 is involved in malignant progression associated with ST6GALNAC4. As previously described orthotopic liver model were used. In shst6galnac4 hep1-6 group, we observed dramatic reduction of galectin3+ TAMs but no significant change in macrophages in spleen (Fig. 7A, B). We infected macrophages with an adeno-associated virus (AAV) expressing shgalectin3 (pAAV-CD68p-EGFP-MIR155(MCS)-WPRE-SV40 PolyA). As a control group, mice were injected with AAV expressing shNC. Macrophages in spleen were isolated for detecting the efficiency of AAV infection. The ratio of CD8+ T cells at tumor were significantly higher in galectin3-RNAi group compared with control (Fig. 7C, D). Likewise, we knocked down galectin3 using shRNA constructs in immortalized murine macrophages (RAW264.7). we tested functions of galectin3+macrophages in a tumor model of subcutaneous established on C57BL/6 mice through inoculation of Hepa1-6 cells and macrophages (at ratio of 2:1,shNC vs. shgalectin3). The result further confirmed our previous results showing a key role for galectin3+ TAMs in mediating HCC growth (Fig. 7G, H). Benzyl-$\alpha$-GalNAc were then used to inhibit the synthesis of the T-antigens, the results of followed flow cytometry analysis showed that the proportion of galectin+3 macrophage is greatly reduced in the inhibitor group (Additional file 7: Fig. S4E) All these proved the galectin3+ TAMs play cancer-promoting role mediated by ST6GALNAC4.

We next analyzed effects of galectin3 inhibitor GB1107, as a galectin3 inhibitor, had been shown to decrease galectin-3 protein levels [24]. We used Hepa1-6 cells expressing the fluorescence to build orthotopic liver model. In our study, GB1107 were injected intraperitoneally once daily 10 mg/kg from the second day post-inoculation. The ability of GB1107 to inhibit HCC tumor growth was further confirmed using fluorescent images taken by the in vivo imaging system (IVIS) (Fig. 7E, F).

## Discussion

In our study, we stratified HCC patients from the database of The Cancer Genome Atlas (TCGA) by gene expression of glycosyltransferase related to O-glycosylation. Based on our analysis, samples belonging to different groups might express different O-glycosylation levels. Overall survival difference for the two groups revealed that high O-glycosylation levels might correlate with tumor progression and poor prognosis. Combining the prognostic information in TCGA and existing literature, we decided to focus our inquiry on ST6GALNAC4 which might increase HCC GalNAc-linked glycosylation as an oncogene. Few studies have examined the molecular function of ST6GALNAC4. Our study indicated that ST6GALNAC4 has a cancer-promoting role in HCC In vitro and in vivo functional warranting further mechanistic study.

The samples from TCGA were clustered with ConsensusClusterPlus following enrichment analysis. TGF-$\beta$ signaling were considered top enriched. TGF-$\beta$ signaling pathway was previously shown to play a variety of roles in HCC [25–28]. Transforming growth factor-$\beta$s (TGF-$\beta$s), bone morphogenic proteins (BMPs), activins, ligands could form dimers which bind type I and type II receptors, activate both SMAD-dependent and -independent pathways, and associated with epithelial-to-mesenchymal transition [29, 30]. Several studies have recently demonstrated glycosylation of multiple proteins in the TGF-$\beta$ pathway can regulate TGF-$\beta$ signaling [18]. Regarding classical signaling receptors, glycosylation can affect multiple functions. Core fucosylation of TGFBR1 and TGFBR2 have an important role in receptor binding [31]. Other studies have shown that N-linked glycosylation of TGFBR2 have a significant impact in transporting to the surface and sensitivity to TGF-$\beta$ [32]. Glycosylation changes always correlate with modification by glycosylation enzymes. Deletion of FUT8, which catalyzes core fucosylation of *N*-glycans, reduced activation of TGF-$\beta$ signaling pathways in renal tubular cells [33]. This mechanism of action is also seen in tumors [34]. MGAT5, which catalyzes branching of *N*-glycans, could promote sensitivity of TGF-$\beta$ signaling [35]. FUT3 and FUT6, involved in glycosylated antigen synthesis, is related to fucosylation of TGFBR1 and regulation of the receptors’ activation [36]. These previous studies lead to the conjecture that whether ST6GALNAC4 change the states of glycosylation of receptors to promotes tumor aggression. Experiments we next performed found ST6GALNAC4 really interact with TGFBR2. GalNAc modification in TGFBR2 was also observed to be positively correlated with ST6GALNAC4. The positive correlation between ST6GALNAC4 and TGFBR2 provided experimental evidence for above possibility as well.

Compared with *N*-glycosylation, O-glycosylation had been studied in fewer studies. There are several types of O-glycans, such as O-Fuc, O GalNAc, and O-GlcNAc [37]. One of the most common types of protein glycosylation is called mucin-type O-glycosylation that is initiated via GalNAc to serine or threonine [7, 8]. This structure of GalNAc to Ser/Thr with an $\alpha$-linkage is called Tn antigen (GalNAc$\alpha$1-O-Ser/Thr, CD175). The structure can be further extended by the addition of Gal, GlcNAc, or GalNAc to the 3-hydroxyl and/or 6-hydroxyl groups to form T antigen, Thomsen-Friedenreich, or TF antigen (Gal$\beta$1-3GalNAc$\alpha$1-O-Ser/Thr, CD176) or other structure [38]. Abnormal glycosylation in numerous cancers has been widely confirmed [39, 40]. As the most common tumor-associated glycan modifications, truncated O-glycans (T- and Tn-antigen) are associated with tumor malignancy in pancreatic cancer [41], melanoma [42], and numerous other cancers [43]. Cao et al. [44] have demonstrated the presence of T antigen in HCC, which lack an in-depth study. In this study, we identified ST6GALNAC4 related high T antigen expression in HCC. We also found evidences of a novel mechanism involving the T antigen, and its role in galectin3+ macrophages recruitment in HCC. Our study provides one such possibility that galectin3 inhibitors might be an acceptable treatment choice for HCC patients with high T antigen expression.

## Supplementary Information

**Additional file 1: Table S1.** Demographic and clinical characteristics of included patients.

**Additional file 2.** Code.

**Additional file 3.** Supplementary methods.

**Additional file 4: Fig. S1.** The association between candidate genes and prognosis in HCC.

**Additional file 5 : Fig. S2.** The immunohistochemical evaluation criteria.

**Additional file 6: Fig. S3.** Overexpression of ST6GALNAC4 enhanced HCC cell proliferation, migration, and invasion in vitro.

**Additional file 7: Fig. S4.** T antigen was highly upregulated in HCC. ST6GALNAC4 expression correlated with T antigen in HCCLM3 cells. Proportions of galectin3^+^ TAMs  populations in control group and O-galnac inhibitor group.

## Data Availability

The data sets supporting the conclusions of this article are included within the article.
