# Supplementary material for: ST6GALNAC4 promotes hepatocellular carcinogenesis by inducing abnormal glycosylation
Source: J Transl Med. 2023 Jun 29;21:420. doi: 10.1186/s12967-023-04191-7 (PMC10308692; doi:10.1186/s12967-023-04191-7)
Supplement: Supplementary file 1 — Additional file 1: Table S1. Demographic and clinical characteristics of included patients. [file 12967_2023_4191_MOESM1_ESM.docx]

| Variables | ST6GALNAC4 expression | | p-value |
| --- | --- | --- | --- |
|  | low | high |  |
| age |  |  |  |
| ≤50 years | 21 | 19 | 0.3318 |
| >50 years | 28 | 22 |  |
| Gender |  |  |  |
| Female | 7 | 3 | 0.2948 |
| Male | 42 | 38 |  |
| HbcAb |  |  |  |
| Negative | 3 | 4 | 0.4945 |
| Positive | 45 | 35 |  |
| HBsAg status |  |  |  |
| Negative | 10 | 9 | 0.8979 |
| Positive | 38 | 32 |  |
| Cirrhosis |  |  |  |
| Absent | 3 | 6 | 0.191 |
| Present | 45 | 35 |  |
| Tumor encapsulation |  |  |  |
| Complete | 23 | 19 | 0.882 |
| No complete | 25 | 22 |  |
| AJCC stage |  |  |  |
| Stage I | 32 | 31 | 0.2881 |
| Stage II & III | 17 | 10 |  |
| Recurrence |  |  |  |
| No | 23 | 18 | 0.7733 |
| Yes | 26 | 23 |  |
| Tumor number |  |  |  |
| Single | 45 | 34 | 0.1987 |
| Multiple | 4 | 7 |  |
| AFP |  |  |  |
| ≤ 400 μg/L | 29 | 28 | 0.4402 |
| >400 μg/L | 19 | 13 |  |
