# Supplementary material for: ST6GALNAC4 promotes hepatocellular carcinogenesis by inducing abnormal glycosylation
Source: J Transl Med. 2023 Jun 29;21:420. doi: 10.1186/s12967-023-04191-7 (PMC10308692; doi:10.1186/s12967-023-04191-7)
Supplement: Supplementary file 3 — Additional file 3. Supplementary methods. [file 12967_2023_4191_MOESM3_ESM.doc]

**Supplementary methods.**

1.Immunohistochemistry (IHC)

Immunohistochemistry (IHC) was performed on paraffin-embedded tissues using a two-step method of Dako Envision™ Detection System (DakoCytomation, Glostrup, Denmark). The antibodies used are listed below：ST6GALNAC4(AB127016) from abcam; PCNA (GB11010

) from Servicebio; KI67 (GB111499) from Servicebio; CD8 (GB13429) from Servicebio; peanut agglutinin (PNA)(L7759) from Sigma-Aldrich.

An IHC index (range of scores, 0 to 12) is defined as the product of the intensity and percentage of cells staining: 0(weak), 1 (medium), 2 (strong) and 3 (very strong). Percentage of positive cells was scored as: 1 (0–25%), 2 (26–50%), 3 (51–75%) and 4 (>75%). Overall score was determined by multiplying the intensity score by positive cell percentage score. Overall score more than 6 (≥6) was defined as high expression, whereas the others represented low expression. The slides were analyzed by two independent pathologists, blinded to clinical outcomes.

2.Cell culture and reagents

Human HCCLM3 and MHCC97H were obtained from the Cell Bank of the Shanghai Institutes of Biological Sciences, Chinese Academy of Sciences. Mouse Hepa1-6 cells were obtained from America Type Culture Collection (Manassas, VA). All cell lines were maintained in Dulbecco's Modified Eagle Medium (DMEM, Life technologies, 6 Gaithersburg, MD) supplemented with 10% FBS, 1% penicillin/streptomycin solution.

3.Western blotting (WB)

Ice-cold radioimmunoprecipitation assay (RIPA) buffer containing protease and phosphatase inhibitors were used to extracte proteins from cell pellets. The proteins were separated by Sodium dodecyl sulphate-polyacrylamide gel electrophoresis (SDS-PAGE). Separated proteins were transferred to polyvinylidene fluoride membranes and blocked in 5% milk in Tris-buffered saline, with 0.05% Tween-20, and incubated overnight with primary antibodies at 4 °C. Quantification of band intensities were quantified by Image Lab software (Bio-Rad, Hercules, CA, USA). TGFB1 protein (HYP7118) was obtained from MedChemExpress (MCE, USA). Primary antibodies for WB included: ST6GALNAC4(AB127016) from abcam; TGFBR2(A11765) from ABclonal; E-cadherin(A20798) from ABclonal; N-cadherin(A19083) from ABclonal; β-Tubulin(A12289) from ABclonal; Peanut agglutinin (PNA)(L7759) from Sigma-Aldrich; Anti-O-GalNAC(SAB5202328) from Sigma-Aldrich. SMAD2/3 (A7536) from ABclonal; pSMAD2 (AP1007) from ABclonal; pSMAD3 (AP0727) from ABclonal; Primary antibodies for Co-immunoprecipitation included: ST6GALNAC4(A12880) from ABclonal; TGFBR2(A11765) from ABclonal.

4.RNA interference

Sequences of siRNAs used in this study are as follows: ST6GALNAC4#1 sense（5'-3'): GCGCAACUAUUCACACUACUUdTdT, antisense（5'-3') AAGUAGUGUGAAUAGUUGCGCdTdT; ST6GALNAC4#2 sense（5'-3'): GGAUGGUCAGCGACAGCUAdTdT, antisense（5'-3') UAGCUGUCGCUGACCAUCCdCdA.

Cells were transfected with siRNAs using jetPRIME (PolyPlus). The cells were cultured for 48 h before phenotypic and functional testing. We purchased ST6GALNAC4 siRNA fromShangya Biotechnology (Hangzhou, China). Transfection efficiency was measured via qRT-PCR and WB analysis.

5.stable cell clone establishment

The targeting sequence of shRNA is as follows: m-shRNA-st6ganac4: GCACCGGTTGGTTCACCATGA; h-shRNA-ST6GALNAC4: GGATGGTCAGCGACAGCTA; m-LGALS3-shRNA-1: GGAGAGTCATTGTGTGTAA: m-LGALS3-shRNA-2: ACACGAAGCAGGACAATAA; All the shRNA and plasmids were purchased from REPOBIO (Hangzhou, China). To produce lentivirus, The lentiviral vectors or plasmids were co-transfected with helper plasmids into 293T cells. The viral supernatant was collected 48 hours after transfection and was directly used to infect target cells. Infected cells were drug-selected using 3 μg/ml puromycin.

6.Cell proliferation assay

Proliferation assays were performed using the Cell Counting Kit-8 (CCK-8 Dojindo Laboratories, Kumamoto, Japan) according to manufacturer's instructions. Cells (2*103 cells per well) were seeded in 96-well clear bottom plates and incubated for 1h at 37 °C. The absorbance was read at 450 nm. For colony formation assay, cells were seeded in duplicate in 6-well plates (2*103 cells/well). Colonies were counted after 10–14 days of incubation at 37 °C.

7.Migration and invasion assays

A transwell in vitro invasion assay was used to quantify and characterize the cell migration and invasion ability of HCCLM3 and MHCC97H cell. Cells were seeded into the upper chamber of a Transwell insert (6.5 mm diameter, 8 μm pore size; Corning) in serum-free medium coated with Matrigel® Basement membrane matrix (Corning) for invasion assays, and non-coated for migration assays. After incubation, the number of migrating or invading cells in the bottom chamber was calculated.

7.Wound healing migration assays

The two wells of the two-chamber moulds were transferred into individual wells of a 12-well plate. Cells were suspended in serum-free media and seeded into the inner chamber (5 × 10^4^ cells per chamber). The percentage was calculated using the following equation: [Wound area (0h) −wound area (48h)] ×100/wound area (0h) = % wound closure.

9.Animal model and treatment

For xenograft model, 5 × 10^6^ HCCLM3 or MHCC97H cells suspended in 100 μl PBS were subcutaneously injected into the flanks of nude mice. Tumor sizes were measured every 7 days using digital calipers, and tumor volumes were calculated using the following formula: volume = 0.5 × (length × width2). Four weeks after the injection, tumors were surgically dissected from the mice, weighed, fixed in formaldehyde, and embedded in paraffin for histology. For orthotopic tumor model, 5 × 10^5^ hep1-6 cells in 12.5ul of PBS were injected into left liver lobe of male C57BL/6 mouse (6 weeks old) mixed into 12.5ul of Basement Membrane Matrix (354248, Corning, Matrigel) to establish the orthotopic model. After sacrificing the mice, tumors were removed and weighed.For orthotopic liver model builded by Hepa1-6 cells expressing the fluorescence, vivo imaging system (IVIS) were used to measure the fluorescence value every 3 days. For RAW264.7 cell and Hepa1-6 cell mixing experiment, the volumes of the subcutaneous tumours were measured every 2 days. For the lung metastasis model, 2× 10^6^ HCCLM3 cells suspended in 100μl PBS were injected through the tail vein. For adeno-associated virus (AAV) infecting model, AAV vectors were administered intravenously via the tail vein. Galectin-3 inhibitor GB1107(HY-114409) was obtained from MedChemExpress (MCE, USA). Benzyl-*α*-GalNAc (HY-129389) was obtained from MedChemExpress (MCE, USA). All animal experiments were approved by the Ethics Committee for Laboratory Animals of the First Affiliated Hospital of Zhejiang University.

10.Flow cytometry

# Mice were sacrificed 10-12days after tumour inoculation and the tissues were dissected out. Dissected tumor or spleen were minced and single-cell suspensions prepared by passing the sample through a 70-μm cell strainer. Tumor-infiltrating mononuclear cells were isolated by OptiPrep Density Gradient Medium (Sigma-Aldrich, St. Louis, MO). Multicolored flow cytometry analyses were performed on Canto II (BD, China). The antibodies were listed as follows: PerCP/Cyanine5.5 anti-mouse CD45(103132); PE/Cyanine7 anti-mouse CD3(100220); FITC anti-mouse CD8a (100706); PE anti-human/mouse Granzyme B Recombinant (372208); APC anti-mouse Perforin (154304); PE anti-mouse CD45(103106); APC anti-mouse CD3ε (100312); PE/Cyanine7 anti-mouse CD4(100422); APC anti-mouse INF-γ(505809); PE anti-mouse TGF-α(506305) ; APC anti-mouse/human CD11b(101212); PE/Cyanine7 anti-mouse/human Mac-2 (Galectin-3)(125417). APC-Cy7 CD45(103116); BV786 CD3(100232); BV650CD11b(101239); BV711CD11c(117349); PerCP-Cy5.5 LY6G(127615); APC CD206(141708); PACIFIC BLUE LY6C(128014); PE F4/80(111704); UV395 IA/IE(107669); FITC CD45(157608); PE CD25(113704); PerCP-Cy5.5 CD3(100218); PE-Cy7 CD4(116016); Alexa Fluor 488Foxp3(320029); PE-Texas Red NK1.1(108748); Above antibodies were purchased from Biolegend.

11.Immunofluorescence

Immunofluorescence was performed on tissue sections directly on slides or cell seeded on slides. The antibodies were listed as follows: Peanut agglutinin (PNA)(L7381) from Sigma-Aldrich; Anti-O-GalNAC(SAB5202328) from Sigma-Aldrich. ST6GALNAC4(A12880) from ABclonal; TGFBR2(A11765) from ABclonal; TGFBR2(GB14153) from Servicebio; CD11b (GB11058) from Servicebio; galectin3 (AB776245) from abcam; CD68 (GB113109) from Servicebio.

**Supplemetary Figure legend**

FigS1 The association between candidate genes and prognosis in HCC. KM curves of ADAMTS1(A), ADAMTS5(B), ADAMTS10(C), ADAMTSL2 (D), B3GNTL1(E), GALNT8(F), GALNT16(G), GALNT17(H), MUC12(I), POMGNT1(J), THSD4(K), THSD7B(L) in TCGA data sets.

FigS2 The immunohistochemical evaluation criteria. (A)Immunohistochemical score of 0 (weak), 1 (medium), 2 (strong) and 3 (very strong).

FigS3 Overexpression of ST6GALNAC4 enhancered HCC cell proliferation, migration, and invasion in vitro. We used plasmids to establish the stable ST6GALNAC4 overexpression cell lines. ST6GALNAC4 overexpression resulted in the enhancing proliferative capacity of HCCLM3(A) and MHCC97H(B) detected by CCK-8 and colony formation assay. ST6GALNAC4 overexpression resulted in higher migratory and invasive capabilities of HCCLM3(C, E) and MHCC97H (D, F). scale bar, 200 μm; magnification, 100X. *P < 0.05; **P < 0.01; ***P < 0.001; ****P < 0.0001. ns, not significant.

FigS4 T antigen was highly upregulated in HCC (A) IHC staining of T antigen in HCC and adjacent liver tissue (scale bar, 50 μm; magnification, 400X). (B) Immunofluorescence of T antigen in HCC and adjacent liver tissue (scale bar, 100 μm; magnification, 200X). (C) Western blotting of T antigen using PNA in HCC and adjacent liver tissue. (D) ST6GALNAC4 expression correlated with T antigen in HCCLM3 cells. (E)Proportions of galectin3^+^ TAMs populations between Benzyl-α-GalNAc group and control group on flow cytometry.
