# Supplementary figures and images for: ST6GALNAC4 promotes hepatocellular carcinogenesis by inducing abnormal glycosylation
Source: J Transl Med. 2023 Jun 29;21:420. doi: 10.1186/s12967-023-04191-7 (PMC10308692; doi:10.1186/s12967-023-04191-7)

**FigS1****A**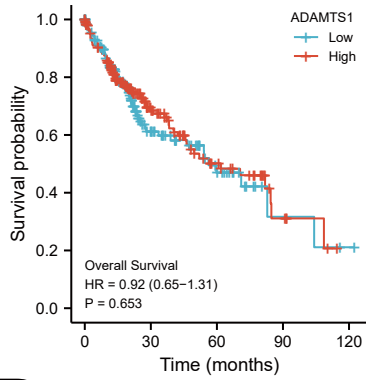**B**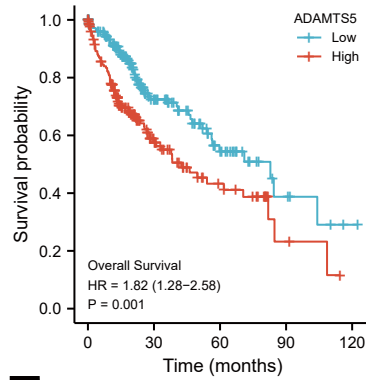**C**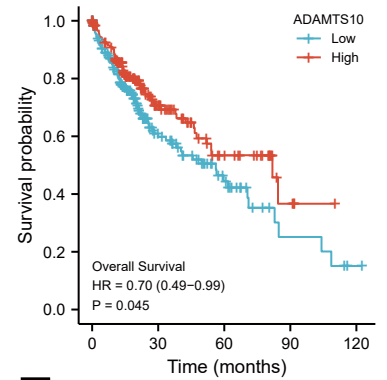**D**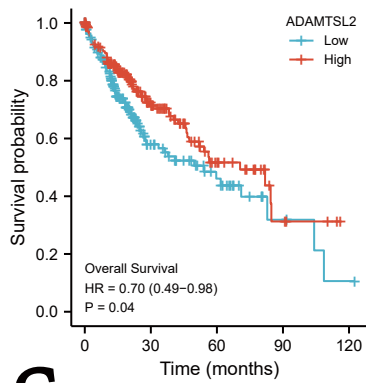**E**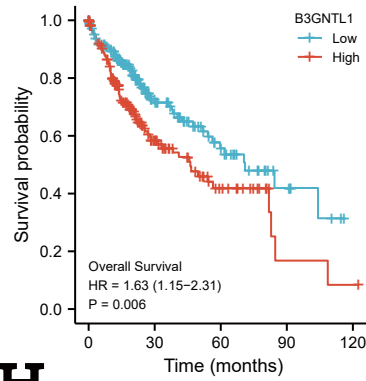**F**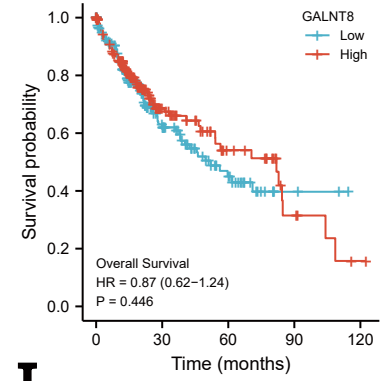**G**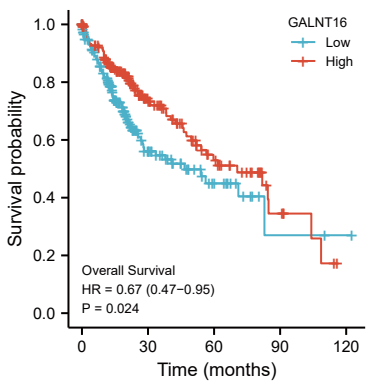**H**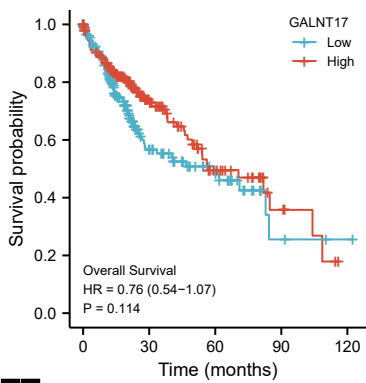**I**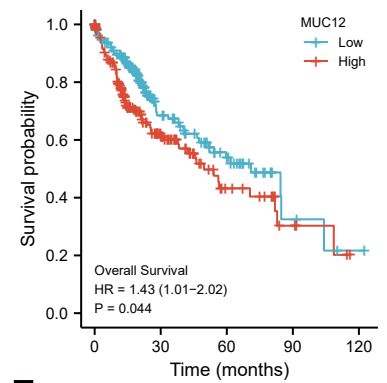**J**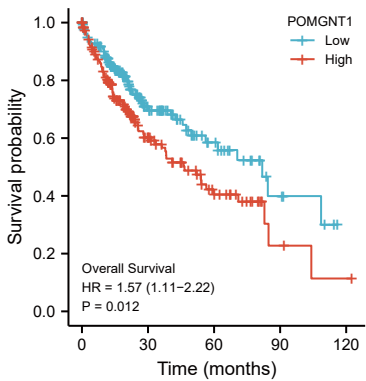**K**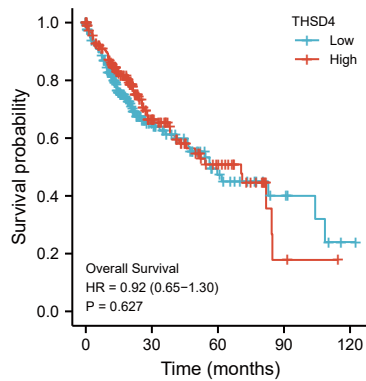**L**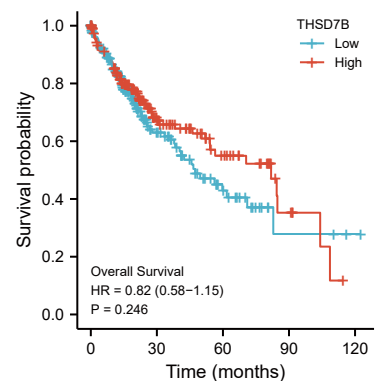

Supplement: Supplementary file 4 — Additional file 4: Fig. S1. The association between candidate genes and prognosis in HCC. [file 12967_2023_4191_MOESM4_ESM.pdf]

FigS2

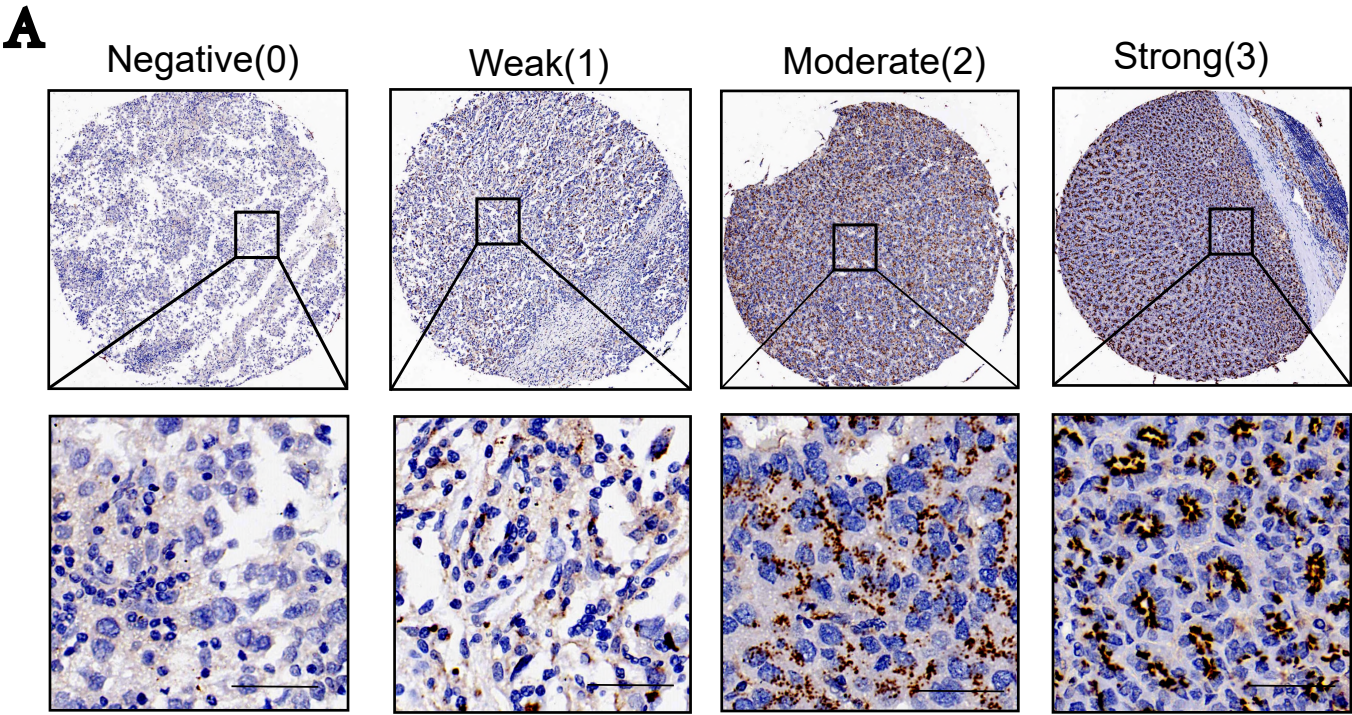

Supplement: Supplementary file 5 — Additional file 5 : Fig. S2. The immunohistochemical evaluation criteria. [file 12967_2023_4191_MOESM5_ESM.pdf]

FigS3

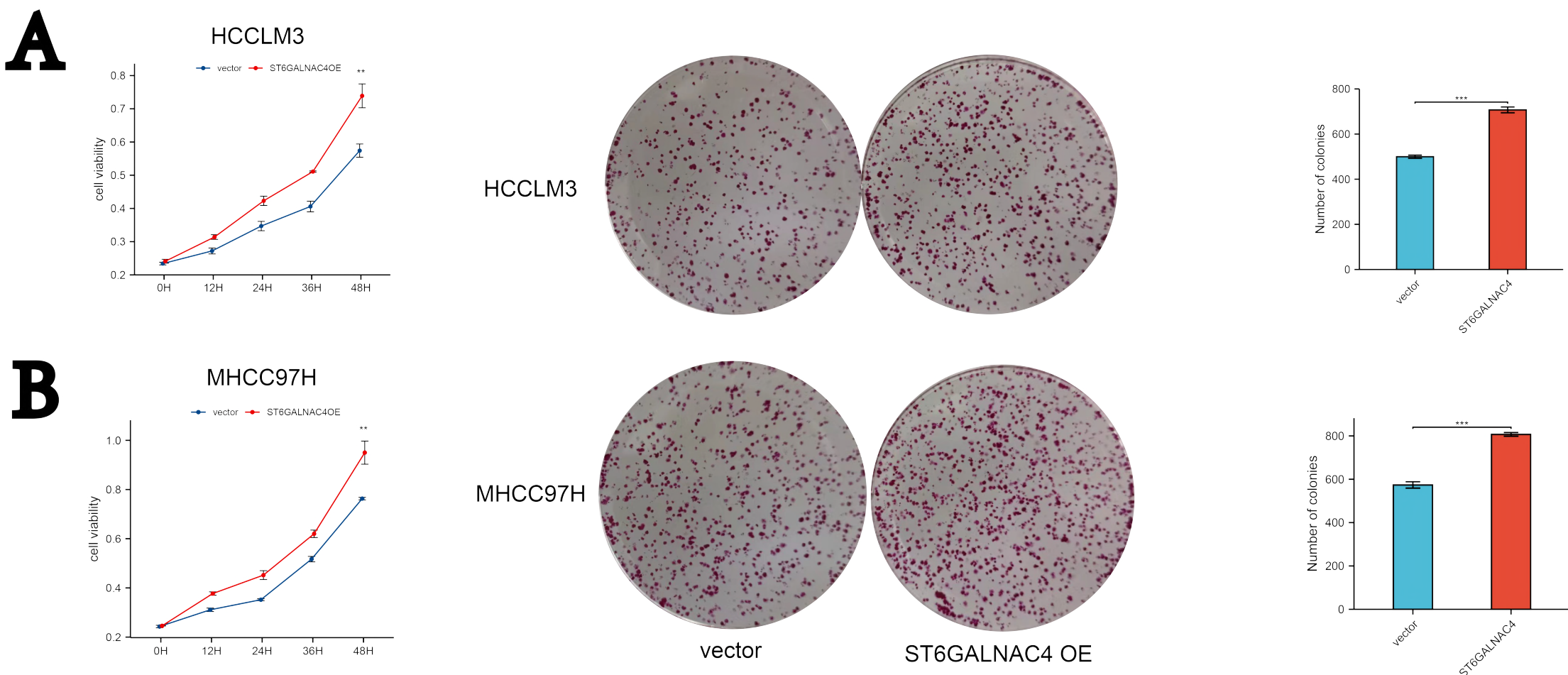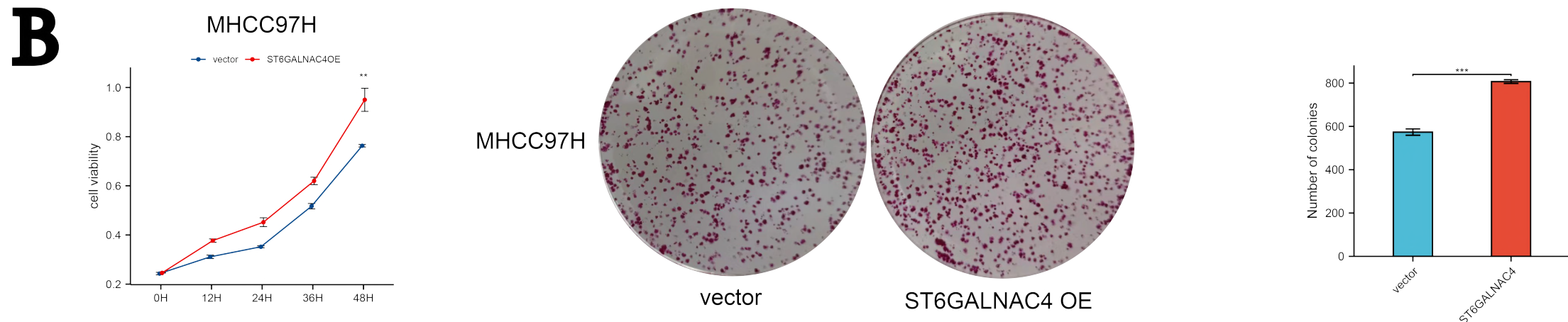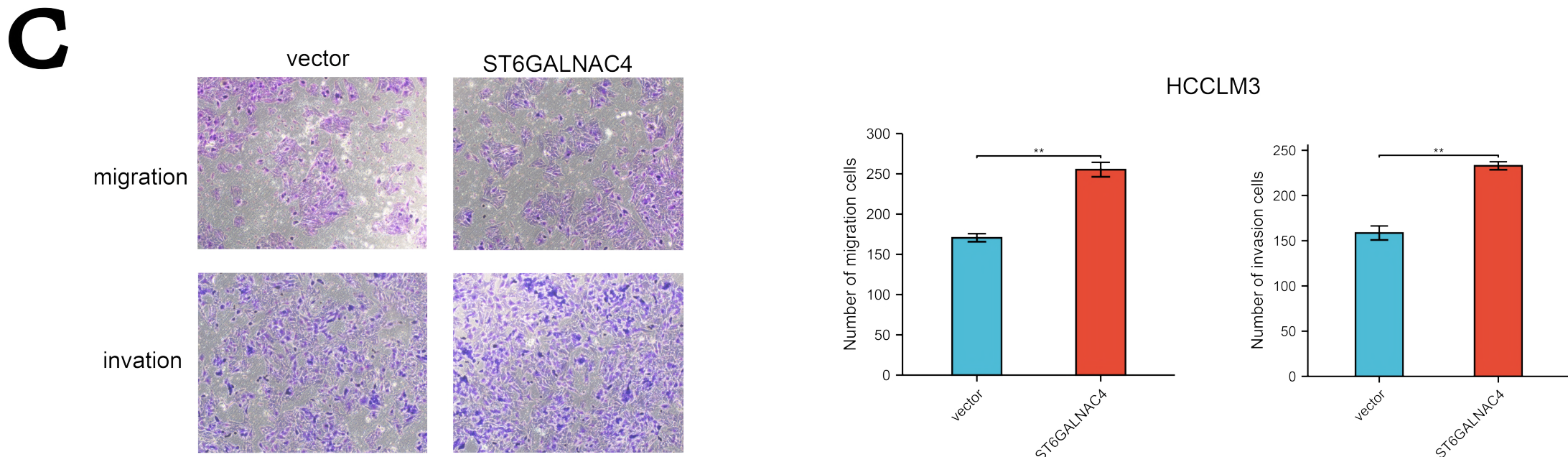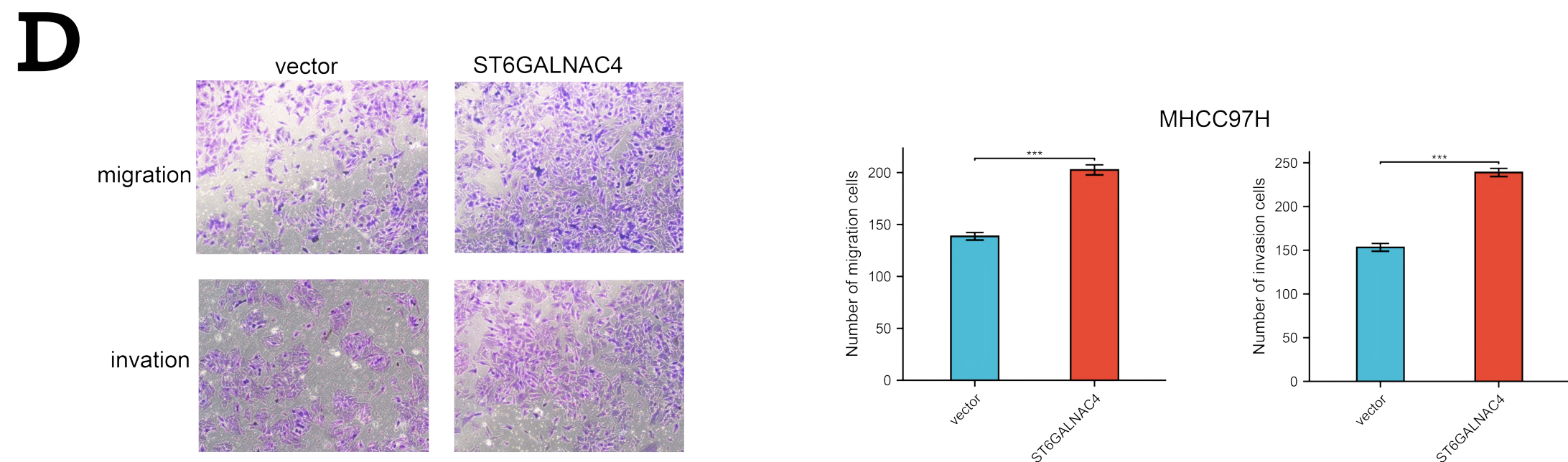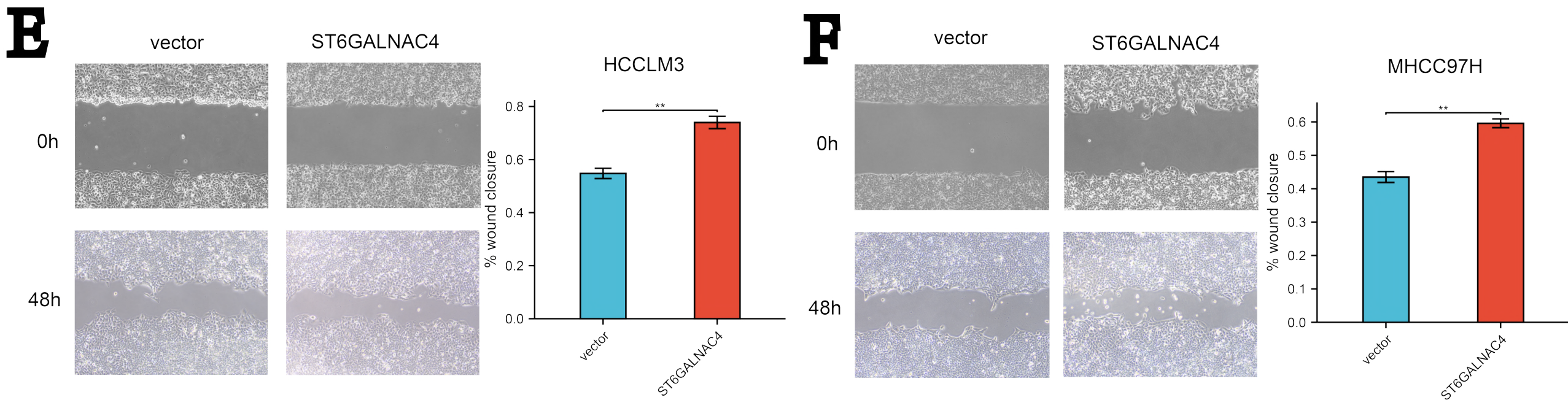

Supplement: Supplementary file 6 — Additional file 6: Fig. S3. Overexpression of ST6GALNAC4 enhanced HCC cell proliferation, migration, and invasion in vitro. [file 12967_2023_4191_MOESM6_ESM.pdf]

**FigS4**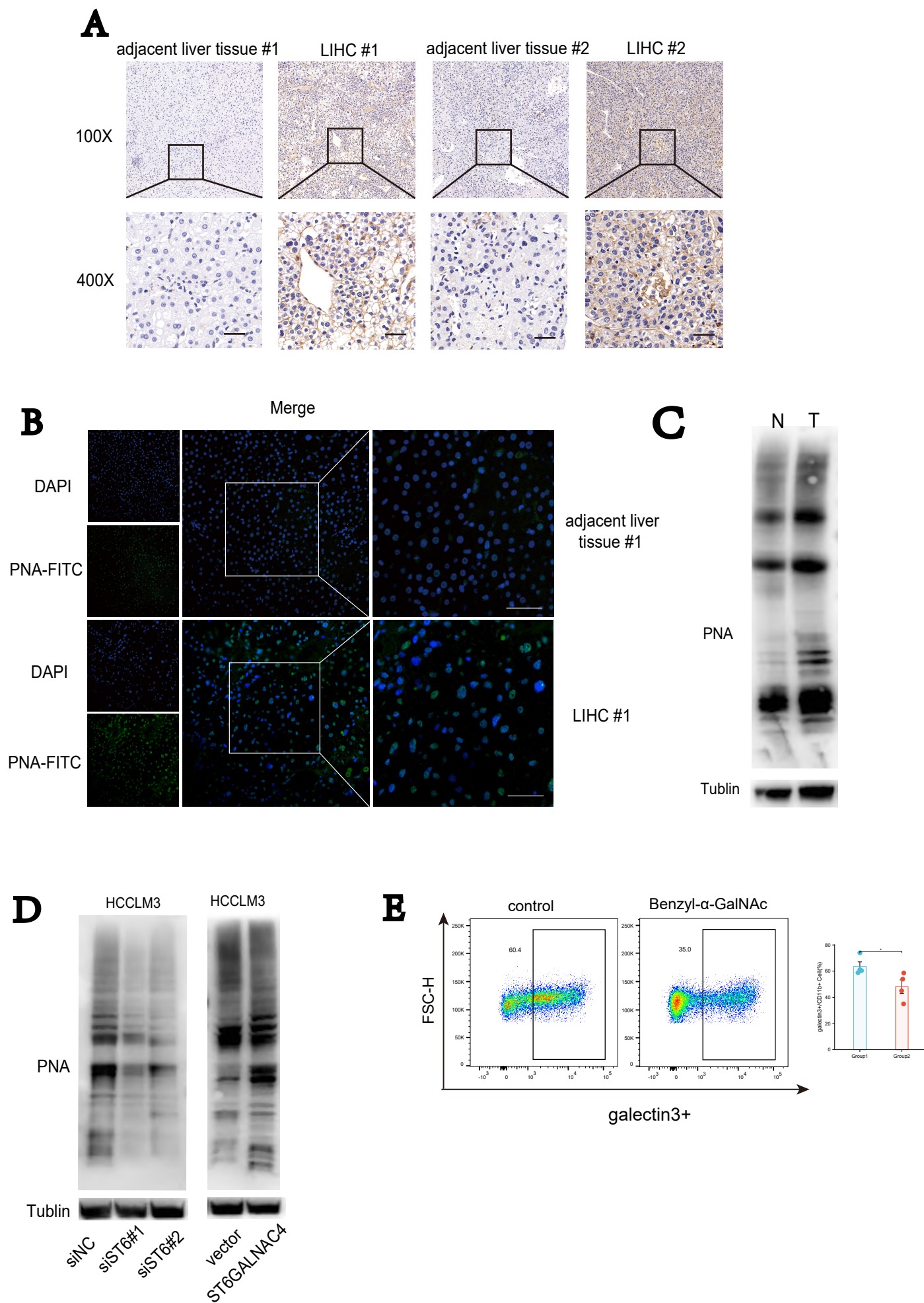

Supplement: Supplementary file 7 — Additional file 7: Fig. S4. T antigen was highly upregulated in HCC. ST6GALNAC4 expression correlated with T antigen in HCCLM3 cells. Proportions of galectin3+ TAMs populations in control group and O-galnac inhibitor group. [file 12967_2023_4191_MOESM7_ESM.pdf]
